# Diet Therapy and Public Health

**DOI:** 10.3390/ijerph19148312

**Published:** 2022-07-07

**Authors:** Zandile June-Rose Mchiza

**Affiliations:** 1 Non-Communicable Disease Research Unit, South African Medical Research Council, Cape Town 7505, South Africa; zandile.mchiza@mrc.ac.za or jmchiza@uwc.ac.za; Tel.: +27-21-938-0673; 2 School of Public Health, University of the Western Cape, Bellville 7535, South Africa

## 1. Introduction

A major threat to the achievement of the Sustainable Development Goals and Universal Health Coverage continues to be malnutrition [1,2]. The term “malnutrition” encompasses both under- and over-nutrition, both of which coexist and interact, especially in low-to-medium-income countries (LMICs) [3]. As a result, the term DBM has been coined [4] to describe this phenomenon, and this is further described as a “malnutrition syndemic” [5] if malnutrition includes micronutrient deficiencies. Research has implicated DBM and syndemic malnutrition in the development of major metabolic diseases, such as coronary heart disease, hypertension, diabetes, and cancer [6]. According to the WHO [7], at a global level, 7 of the 10 leading causes of death in 2019 were metabolic diseases.

Due to the effectiveness of diet therapy in preventing and improving DBM and the malnutrition syndemic [8,9], it appears appropriate to integrate it into all global strategies to prevent and control metabolic diseases [10,11]. A meal plan to control and promote the intake of certain foods or nutrients is called diet therapy [12]. To improve health and well-being, Mudambi and Rajagopal [13] define diet therapy as promoting healthy eating, especially promoting food prescribed by a diet/nutrition specialist. Diet therapy is further defined by Stanfield and Hui [8] as a combination of food items prescribed to cure infectious diseases, gain or lose weight, and prevent or delay chronic diseases. A diet therapy program typically involves modifying a regular diet to meet an individual’s health needs [12]. As part of therapeutics, micronutrients and macronutrients are manipulated to suit the individual’s health needs. The therapeutic diet may be temporal or permanent depending on a person’s health condition [8].

There are some concerns about the aforementioned diet therapy definitions since they somewhat propagandize diet therapy as a “nutricentric” approach, i.e., a treatment that can only be achieved individually [14,15]. This undermines the importance of factors that support diet therapy, such as the food environment, political influence, tradition, culture, and economics. According to Scrinis [15], Traverso-Yepez and Hunter [16], Downs [17], Mozaffarian [18], Bergman [19], Mackenzie [20], and Willett et al. [21], several factors influence food choices, aside from individual dietary practices, nutrition knowledge, age, food preferences, and behavior change. Further, the latest research reveals these as multi-disciplinary and complex approaches that address malnutrition, micronutrient deficiencies, and metabolic diseases, such as food supply, quality, availability, price, labeling, and marketing, that are threaded through policies [17,22,23,24].

Hence, our current Special Issue sought to collect individualistic, comprehensive, and multidisciplinary diet-related public health approaches that are designed to (i) empower the international public with nutrition literacy and dietary behavior change strategies; (ii) improve the food environment; and (iii) consider human rights, tradition, culture, and economic status. We further welcomed diet-related research that highlights the changes that occurred in people’s diets amidst the COVID-19 countrywide lockdown. All these strategies had to be informed by the World Health Organization [25] policy guidelines.

## 2. Diet-Related Public Health Approaches Aimed for This Special Issue

The current Special Issue aimed to cover a wide range of studies that investigated: (i) diet therapies that are prescribed by an accredited professional and their association with body composition and metabolic diseases; (ii) food cost and its association with healthy dietary practices and optimal complementary feeding of children; (iii) food environment and its association with food choice and the nutritional status of adults and children; (iv) social support and food donations such as feeding schemes and their role in achieving good health and food security; and (v) food and nutrition literacy, i.e., presented as food and nutrition knowledge, meal planning, food preparation, food consumption, and the use of food labels amidst COVID-19 countrywide lockdown. In this Special Issue, we also welcomed prototypes of complex, multidisciplinary, and multiphased research aimed at addressing the DBM and malnutrition syndemic and its consequences.

### 2.1. Diet Therapy, Nutritional Status, and Metabolic Disorders

The primary purpose of a diet is to provide energy to power the body. Food energy is derived from macronutrients, i.e., carbohydrates, proteins, and fats. For a healthy diet, 55–65%, 20–30%, and 15% of food energy need to come from complex carbohydrates, essential fats, and proteins, respectively, according to Robinson and Weigley [26] and the World Health Organization [9]. Steyn and Temple [27], Raymond and Morrow [12], and Mudambi and Rajagopal [13] emphasize that a healthy diet should be low in simple sugars, saturated fats, and salt. These diets should be prescribed by an accredited professional for them to be effective.

Increasing efforts are being made to promote sustainable diet therapies that focus on consumer behaviors and contribute to food and nutrition security for present and future generations [17,21,28]. Among these therapies are the Mediterranean, plant-based, DASH, Nordic, low-fat, and high-protein diets [11]. Of note are diets that emphasize the consumption of fruit and vegetables, nuts, whole grains, and legumes [29]. This is on the basis that while these diets are environmentally friendly [28], they also lower the risk of coronary artery disease and stroke [29,30,31,32]. Several beneficial nutrients also contribute to the protective effects of these diets, including mono- and polyunsaturated fatty acids, antioxidant vitamins, minerals, phytochemicals, fiber, and amino acids [33,34,35,36,37]. In diets high in fiber, the glycemic index is low, which helps keep blood sugar levels stable, improve weight loss, and control Type 2 diabetes [30,32,38]. As a general rule, animal proteins contain a full and sufficient balance of essential amino acids needed for bodily functions, thereby being considered ‘high biological value’. However, raising livestock is environmentally unfriendly [28], and animal food products are high in total and saturated fats, including cholesterol [39], which contributes to obesity [30,40] and heart diseases [41,42]. Accordingly, the American Heart Association [29], the Food and Agriculture Organization (FAO) [41], and the WHO [42] recommend the choice of whole grains, lean and plant-based proteins, a variety of fruits and vegetables, and vegetable oils, avocados, nuts, and seeds, as these foods are associated with a lower risk of dying from coronary heart disease and other metabolic diseases.

### 2.2. Diet Therapy and the Cost of a Healthy Diet

Food and nutrition insecurity has far-reaching health and developmental consequences that become evident across the life course [43]. More importantly, reaching nutrition adequacy and leading a long and healthy life may be situations that are farfetched for those individuals who are financially constrained [44]. For instance, the high unemployment rate coupled with a lack of income has a devastating impact because it prevents the poor from procuring good-quality food. Hence, they tend to purchase and consume food that has a high level of saturated fat, refined sugar, and salt [43]. Consuming this food has negative health consequences, among them being the DBM, syndemic malnutrition, and metabolic diseases [45].

In recent years, the COVID-19 pandemic has also reshaped our society’s living conditions such that a majority of households are grappling with financial difficulties, especially in LMICs [44,46,47,48,49,50,51]. In these households, people rely on social security grants as their sources of income. Receiving social security grants does not guarantee an average and basic healthy food basket, given the high cost of a healthy diet [27,46,52,53]. As such, some governments internationally have devised mitigation strategies to improve healthy diet access to their citizenry. Among these are social security grants, food handouts, and feeding schemes afforded to households and individuals that are grappling with financial constraints [54,55,56].

Food cost is also determined by the food outlet selling that specific food. Buying food from supermarkets may give communities the freedom to choose healthy options. However, this includes an added cost, as community members may need to commute to get to these food outlets. Barriers such as financial constraints and lack of access to transport often prevent consumers from shopping outside of their residential areas [55,56]. Hence, they prefer to use convenient community shops such as spaza/tuck-shops and street food vendors [57,58], which mostly sell unhealthy food [59].

### 2.3. Food Environment and Food Choice

The role of food environments in shaping transitioning diets and the DBM in LMICs is increasingly gaining policy attention [60]. The food environment comprises physical, social, economic, and cultural surroundings and opportunities [17]. These factors influence people’s food choices and nutritional status. There is substantial evidence suggesting that the community-level physical food environment contributes to the high levels of obesity and diet-related disease [60,61,62,63,64,65]. Few articles published in LMICs on the topic include studies where it is shown that most of the foods sold in the community convenience stores and by street vendors do not foster good health [57,59]. However, we must be cognizant that the demand by consumers is a key determinant of the food offered by these informal food outlets [58,66]. This is also the case in terms of the type of food sold in schools, as evidence [67,68] has shown that the reason why this food is mostly unhealthy [69] is because children demand unhealthy food [70], and their preference for this food is fueled by food marketing [61]. This has a strong policy implication; hence, policy reforms are required to increase access to affordable healthy food options in communities and schools, so as to curb the indiscriminate sale and marketing of unhealthy food.

### 2.4. COVID-19 Countrywide Lockdown and Food and Nutrition Literacy

The current COVID-19 crisis has magnified the vulnerability of populations across the globe. According to the Committee on World Food Security (WFS) framework developed by the High-Level Panel of Experts [71], all sources of financial income for people in most countries seem to have deteriorated during the COVID-19 countrywide lockdown. The countrywide lockdown is a social distancing policy implemented worldwide to compel citizens to stay away from areas where there are group gatherings to prevent the spread of COVID-19 infections [72]. Among others, these areas include food retail malls, food production places, and food service facilities. The WFS framework also shows that a countrywide lockdown not only results in a disrupted food supply chain but also results in an economic meltdown that, in turn, interrupts the countries’ social protection initiatives, further deepening inequalities within countries. Furthermore, in this framework, it is shown that countrywide lockdown affected the availability, pricing, and quality of food; Barrett [73] defines this disruption as a recipe for the development of poverty and food insecurity. De Baker et al. [74] and Grunert et al. [75] provided international evidence that suggests variations in people’s food literacy and self-reported food consumption in the wake of COVID-19. While some people reported that stay-at-home policies and feelings of having more time during COVID-19 improved their food literacy, others reported a deterioration of food literacy during this time as a product of the lack of access to healthy food. Moreover, in these studies it is highlighted that stress relates to food literacy in more complex ways, thereby highlighting the necessity of policies through a health equity lens.

## 3. Conclusions

The DBM, the malnutrition syndemic, and their associated metabolic diseases are among the leading causes of morbidity and mortality, especially in LMICs. Hence, “diet therapy” is regarded as an integral component of all global strategies to prevent and control these health conditions. There is, however, a high level of polarization surrounding diet therapy, which is viewed as being only achievable on an individual basis. It is important to consider other factors that support diet therapy, including food politics and food environments that are linked to tradition, culture, and economics. The current Special Issue, therefore, sought to compile diverse articles that cover a wide range of studies that considered such diet therapies, including those that (i) promote the prevention and treatment of the DBM, micronutrient deficiencies, and metabolic diseases; (ii) support healthy food choices by supporting the manipulation of food prices and making healthy food available and accessible, especially in communities that grapple with financial constraints; (iii) are culturally and socially sensitive; and (iv) support food environment reforms to consider future pandemics like COVID-19.
